# On the Antimicrobial Activity of Various Peptide-Based Dendrimers of Similar Architecture

**DOI:** 10.3390/molecules20010738

**Published:** 2015-01-07

**Authors:** Tania K. Lind, Piotr Polcyn, Paulina Zielinska, Marité Cárdenas, Zofia Urbanczyk-Lipkowska

**Affiliations:** 1 Nano-Science Center and Department of Chemistry, University of Copenhagen, Copenhagen 2100, Denmark; E-Mail: tania@nano.ku.dk; 2 European Spallation Source ESS A/S, Lund 22363, Sweden; 3 Institute of Organic Chemistry, Polish Academy of Science, Warsaw 01-224, Poland; E-Mails: wormfather@o2.pl (P.P.); paulina.zielinska@icho.edu.pl (P.Z.); 4 Department of Biomedical Sciences and Biofilm Research Center, Health & Society, Malmoe University, Malmoe 20500, Sweden

**Keywords:** dendrimers, peptide, antimicrobial, lipids

## Abstract

Antimicrobial drug resistance is a major human health threat. Among the many attempts to tackle this problem, the synthesis of antimicrobial compounds that mimic natural antimicrobial peptides appears as a promising approach. Peptide-based dendrimers can be designed to have higher potency than natural antimicrobial peptides and at the same time they can evade the bacterial defense system. Novel dendrimers with similar chemical structure but varying potency in terms of minimum inhibitory concentration were designed. The dependency between dendrimer structure and antibacterial activity as well as their capacity to attack model cell membranes was studied. The data suggests that supramolecular structure in terms of charge distribution and amphiphilicity, rather than net charge, is the main driver for disruption of cellular membranes and this correlates well with dendrimer hemolytic activity.

## 1. Introduction

The search for alternative antibiotics to eradicate resistant microbial strains is becoming a worldwide need. In this context the non-specific mode of action of natural amphiphilic antimicrobial peptides [1] is a great source of inspiration. During the last 30 years, extensive studies on the mechanism of action of antimicrobial peptides on cellular membranes have led to the conclusion that membrane binding and destabilization is a key step common for these types of antimicrobial compounds [2,3,4,5,6]. Another way to break antibiotic resistance is to design amphiphilic peptides with a branched structure [7,8,9], *i.e*., peptide-based dendrimers, in order to enhance antimicrobial potency and at the same time overcome degradation by the enzymatic systems of bacteria [10].

Antimicrobial peptides and peptide-based dendrimers have similar structural features: multiple positive charges and an amphipathic structure. The molecular mechanisms by which dendrimers exert their activity are still not fully understood, although they clearly involve interactions with lipid membranes [11]. Quartz crystal microbalance with dissipation (QCM-D) was used to study the adsorption behavior of the dendrimers on silica surfaces and supported lipid membranes made of 1-palmitoyl-2-oleoyl-*sn*-glycero-3-phosphocholine (POPC) in order to elucidate the relationship between chemical structure and affinity for lipid bilayers. The dendrimers were synthesized around the same core molecule and have the same number of cationic groups, but differ in their location (terminal* versus* inside of each dendritic chain), level of branching (5* vs.* 9 branches) and on the polarity of the terminal residues (Boc-* vs.* 2-Cl-Boc group). These dendrimers belong to a family of cationic amphiphiles and are therefore thought to act mainly by disrupting the cellular membrane [11]. The study of their interactions with model lipid membranes may significantly enhance the understanding of their mechanism of action and explain differences in antimicrobial activity.

## 2. Results and Discussion

### 2.1. Organic Synthesis

**Figure 1:**
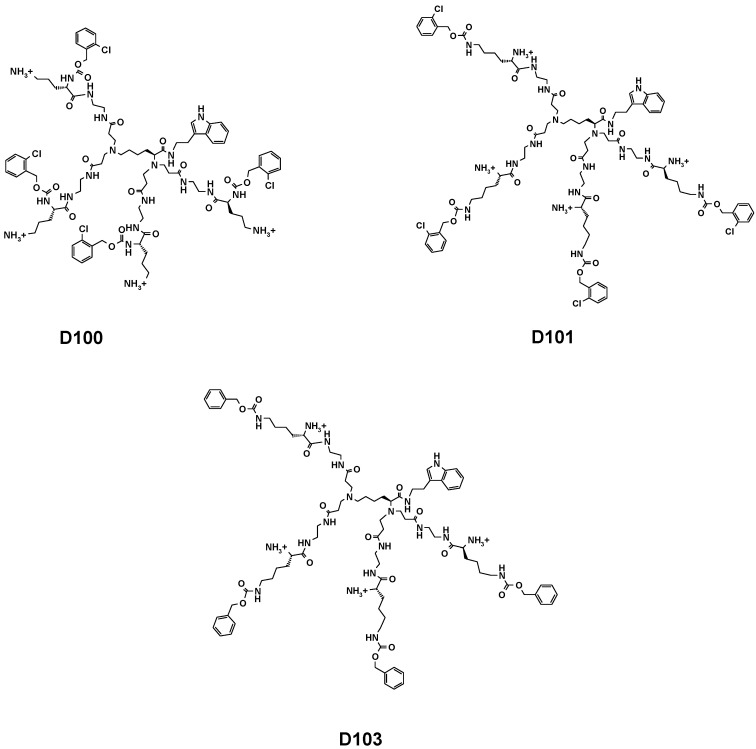
Chemical structures of the studied dendrimers D100, D101 and D103.

The dendrimers used in this study were synthetized according to previously reported procedures [12]. Figure 1 shows their structures. Briefly, the synthesis involves aza-Michael addition of amine groups of l-lysine to methyl acrylate, followed by coupling of the *C*-terminus with tryptamine and then amidation of the resulting methyl esters with a large excess of ethylenediamine. Further coupling reaction of core molecules with orthogonally substituted l-lysine and subsequent Boc-group deprotection afforded dendrimers D100 and D103 as water-soluble hexahydrochlorides. The structure of the dendrimers was confirmed by ESI MS, ^1^H- and ^13^C-NMR techniques. Synthesis and analytical data for dendrimer D101 was reported previously [12].

### 2.2. Antimicrobial and Hemolytic Activity of Dendrimers

Table 1 gives the potency of the dendrimers expressed as MIC values against Gram-positive antibiotic susceptible *Staphylococcus aureus* ATCC 25923, and antibiotic resistant *Staphylococcus aureus* ATCC 43300 as well as antibiotic susceptible *Escherichia coli* ATCC 25922 and *Pseudomonas aeruginosa* ATCC 27853 Gram-negative reference strains. Despite bearing the same (+)6 charge the dendrimers present rather different antimicrobial activities with dendrimer D100 being the most active, followed closely by D101. Moreover, D100 is three times more potent against a Gram-negative *E. coli* strain than D101.

Dendrimer D101 and D103 are structurally similar in terms of the distribution of hydrophobic groups and the charges. However, dendrimer D103 lacks chlorine atoms in the hydrophobic surface residues and this chemical difference makes dendrimer D103 ten times less active against both Gram-positive and Gram-negative strains as compared to D101. Table 1 also shows the hemolytic properties of the dendrimers (HEM), expressed as the dendrimer concentration that induces cell lysis of 100% erythrocytes in relation to a standard. Interestingly, compound D100 is two times less hemolytic than D101, although it presents the same potency against Gram-positive bacteria as D101. The less anti-microbial active compound D103 is also much less harmful against red blood cells.

**Table 1:** MIC values against different bacterial strains and hemotoxicity (HEM) for dendrimers D100, D101 and D103.

Strain	MIC in μM ^(1)^		
	100 (2-Cl-Z)-Lys	101 Lys(2-Cl-Z)	103 Lys(Z)
*S. aureus*, *ATCC 25923*	0.93	0.93	12.9
*S. aureus*, ATTC 43300	5.81	10.0	74
*E. coli*, ATTC 25922	3.71	12.0	111
*P. aeruginosa*, ATTC 27853	41	51	149
HEM (µM)	64	32	≫128 ^(2)^

Notes: ^(1)^ MICs of the reference compounds: penicillin G against *S. aureus* ATCC 25923-6.6 (μM); polymyxin B against *E. coli* ATCC 25922 and *P. aeruginosa* ATCC 27853-0.55 (μM); ^(2)^ at higher concentration the dendrimer precipitates from solution.

### 2.3. Interfacial Behavior at Silica Surfaces

**Figure 2:**
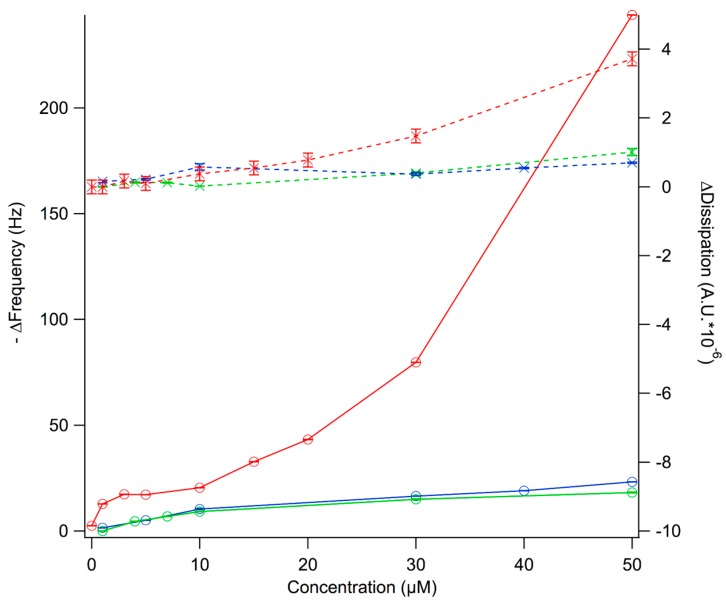
Adsorption isotherms for peptide-based dendrimer D100 (green), D101 (red), and D103 (blue) on silica. ∆f (circles) and ∆d (crosses) are plotted as a function of bulk dendrimer concentration. Lines act as guides to the eye.

QCM-D was used as a method to follow the adsorption of dendrimer solutions to clean silica surfaces and thus to give a relative measure of the affinity of these dendrimers for the negatively charged silica surface. QCM-D measures simultaneously a change in frequency (∆f), which is related to the wet adsorbed mass as well as a change in dissipation (∆d), which gives information on the viscoelastic properties and softness of the layers. For low ∆d, the adsorbed mass (∆m_a_) can be calculated using the Sauerbrey relation; Δm_a_ = −C·Δf, where C = 17.7 ng·cm^−2^·Hz is the material specific Sauerbrey constant for the silicon crystals [13]. The adsorption isotherms were measured by exposing clean silica surfaces to dendrimer solutions of concentrations ranging between 1 and 50 µM in PBS buffer. The results after 1 h incubation are shown in Figure 2, where both −∆f and ∆d are plotted as a function of dendrimer concentration. Steady-state conditions were reached for all concentrations tested within the time frame of the experiment except for dendrimer D101 at concentrations >30 µM. In the latter case, bulk phase separation or the formation of multilayers on the surface took place [12].

For all dendrimers studied, there was an increase in −∆f with concentration until a plateau was reached at ~10 µM for D100 and D103 and at ~3 µM for D101. This is consistent with the formation of a dendrimer “monolayer” on the silica surface. The fact that the adsorbed amount under these conditions was almost doubled for D101 suggests that this dendrimer formed aggregates in solution and adsorbed on silica in the form of dimers or trimers. Increasing the concentration above 10 µM led to a further increase in the adsorbed amount for D101. This is consistent with multilayer formation, which is supported by the fact that no steady state conditions were reached within 1 h of adsorption under continuous flow of dendrimer at concentrations >30 µM. The driving force for dendrimer adsorption to silica does not only arise from the electrostatic forces between the negatively charged surface and the positively charged dendrimers, but also due to an entropic gain as counter-ions are released into the bulk solution upon charge neutralization. The extent of these forces should be comparable for the three dendrimers as they carry the same net charge. However, it is noted that the concentration required to form a full monolayer was considerably lower for D101 and this dendrimer therefore had a higher affinity for silica. This could be a consequence of the formation of stable aggregates of D101 in solution, which then present a higher apparent charge than the monomers. For D101 extensive rinsing with buffer led to mass removal for concentrations above 10 µM, see ref [12]. For all dendrimers, including D101, the adsorbed amount corresponding to monolayer coverage could not be removed with buffer rinsing. This is consistent with irreversible polymer binding to surfaces, where multiple attachment points to the surface create high desorption energy barriers [14].

### 2.4. Interfacial Behavior at Model Cell Membranes

**Figure 3:**
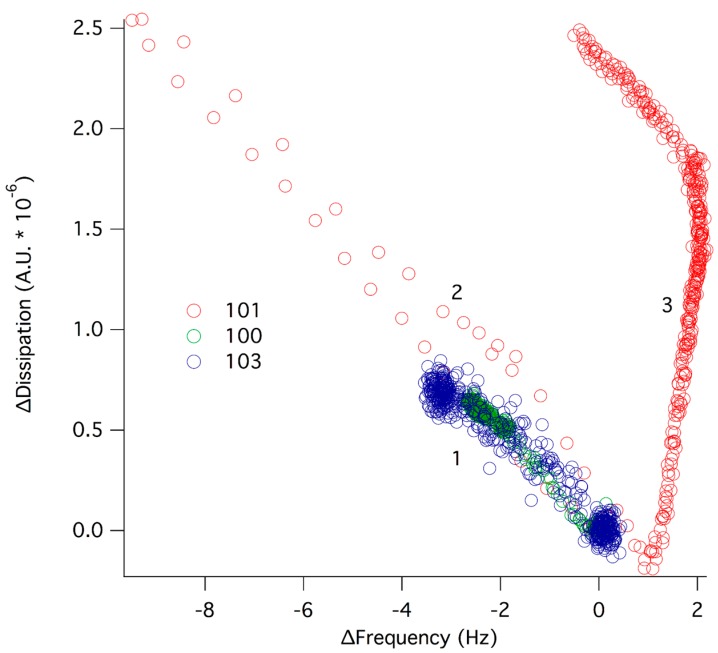
∆d plotted as a function of ∆f for 6 µM of D100 (green), D101 (red) and D103 (blue) interacting with a supported lipid bilayer of POPC. At ∆d, ∆f = 0, dendrimers were added to the supported lipid bilayer. A pure adsorption process (1) occurred for all three dendrimers (increase in ∆d and decrease in ∆f). For D101, the adsorption was followed by desorption (2) and layer rearrangement (3).

Supported lipid bilayers were formed on silica surfaces via fusion of POPC vesicles. The bilayer formation was followed by QCM-D and typical traces comparable to those published previously were observed [15]. The SLB was then exposed to dendrimer solutions at various concentrations. ∆f and ∆d responses were measured over a period of 1 h in which steady state conditions were reached for all dendrimers except D101 at 6 µM. The latter required more than 2 h to reach equilibration. Figure 3 gives a plot of the change in dissipation* versus* frequency for the 7th overtone after exposing POPC to dendrimer solutions of 6 µM concentration. For D100 and D103 a monotonic behavior was observed, where ∆f decreased and ∆d increased, indicating dendrimer adsorption onto the lipid membrane. For D101, on the other hand, the adsorption step was followed by a desorption-dominated regime (increase in ∆f and decrease in ∆d) and then by a complex surface phenomenon where slow desorption with large changes in dissipation occurred. This indicates a major layer rearrangement, as was demonstrated earlier using continuous flow atomic force microscopy [12].

**Figure 4:**
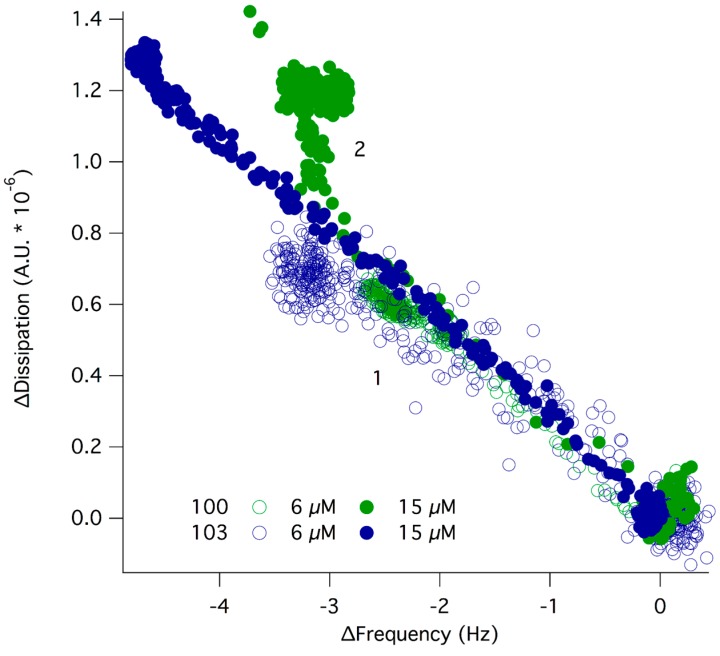
∆d plotted as a function of ∆f for D100 (green) and D103 (blue) interacting with a POPC bilayer at 6 (open symbols) and 15 µM (filled symbols) concentration. At ∆d, ∆f = 0, dendrimers were added to the supported lipid bilayer. Increase in ∆d and decrease in ∆f indicated adsorption of dendrimers to the SLB (1). For D100 at 15 µM, there was a second adsorption regime with increased dissipation pointing to softening of the layer (2).

Interestingly, the initial dendrimer adsorption did not depend on the dendrimer type. However, the layer rearrangement phenomenon was observed exclusively for D101. Thus, the long-range, electrostatic forces that drive the initial dendrimer-lipid binding seem similar for all dendrimers as expected due to their similar charge. However, the relative bulk concentration for monolayer coverage on silica was not comparable between these dendrimers since monolayer coverage was reached on silica at 6 µM for D101 while a concentration of more than 10 µM was needed to reach saturation for D100 and D103 (Figure 2). In order to get comparable surface coverage for the three dendrimers, ∆f and ∆d responses were measured at 15 µM concentration for D100 and D103 (Figure 4). Comparable dendrimer adsorption occurred at this concentration yielding a similar extent of adsorption on the POPC bilayer as for 6 µM, although for 15 µM D100 a second adsorption regime was observed, indicating the formation of a layer with increased dissipation. The latter implies softening of the adsorbed layer.

### 2.5. Antimicrobial Activity, Hemotoxicity, Affinity for Lipid Membranes and Chemical Architecture

From the observations in this work it is clear that dendrimer D101 behaves differently than D100 and D103 on both silica and supported lipid membranes: all dendrimers studied adsorbed to both the silica surfaces (Figure 2) and the POPC supported lipid bilayer (Figure 3 and Figure 4), but only D101 was able to induce rearrangement of the lipid bilayer at concentrations as low as 6 µM (Figure 3 and [12]). This is unexpected given that D100 presents the lowest MIC for all strains tested, closely followed by D101 and then by D103. The latter presents at least ten times higher MIC than D100 for all but *P. aeruginosa* (Table 1). The presence of a chlorine atom in the hydrophobic terminal aromatic rings of D101, combined with the positioning of the charged amino groups inside of the molecule, make this dendrimer prone to self-aggregation at interfaces. This is seen from the increased adsorption beyond monolayer formation on silica observed at higher dendrimer concentrations (Figure 2). Moreover, the net adsorbed amount for the D101 dendrimer (the Sauerbrey adsorbed mass is ~300 ng/cm^2^) on silica was far larger than a monolayer of monomers (~150 ng/cm^2^ assuming they adsorb with the long axis parallel to the surface) suggesting that the D101 dendrimers exist as stable aggregates (dimers/trimers) in the bulk solution at concentrations as low as 6 µM. For the other two dendrimers the net adsorbed amount was below full monolayer formation (~90 ng/cm^2^) at similar concentrations. This suggests that they exist as monomers in the solution at this concentration. If the concentration of D100 and D103 was further increased, the adsorbed amount reached a full monolayer and could even surpass it. This suggests that these two dendrimers could also form aggregates in solution at higher bulk concentration. Furthermore, only dendrimer D101 was able to induce major rearrangements of the POPC lipid bilayer at concentrations at which monolayer formation was observed on the silica surface. Such rearrangements could be due to integration into the lipid bilayer that leads to swelling of the membrane and/or partial solubilization, in a mechanism that resembles surfactant-lipid interactions [16].

Although dendrimer D100 has similar MIC values as dendrimer D101, it is significantly less destructive against POPC membranes. This is in agreement with the lower hemotoxicity of D100 as compared to D101. Upon arrival at the membrane, polar and charged residues of dendrimers are likely located on its water-accessible surface. The adsorbed molecules cover the lipid surface and then most likely rearrange according to a pattern driven by the individual supramolecular properties of the compounds. Electrostatic interactions between the adsorbed dendrimer molecules at the fluid, dynamically changing zwitterionic lipid membrane induce partial charge neutralization, thus allowing for domination of hydrophobic and other van der Waals forces that facilitate the insertion of hydrophobic residues inside the core of the lipid bilayer. To some extent this process resembles 2D organization taking place during liquid crystal formation [17]. For D101 and D103, the charged groups are hidden inside the molecule while there are (+)4 net charge on the D100 surface. Thus, the resulting electrostatic repulsions between molecules probably hinder D100 aggregation on the model membrane. For D101 and D103, the intermolecular interactions of adsorbed molecules on the lipid bilayer are likely dominated by π-π interactions between terminal aromatic systems located at the end of the dendrimer branches. For D101, additional interactions are likely to occur between permanent antiparallel dipoles present in the 2-chlorocarbobenzoxy residues. These additional interactions are enough to push the activity of D101 molecules and allow them to disrupt the structure of POPC bilayers (Figure 3 and ref [12]). Thus, it seems that the supramolecular properties of the molecules are crucial for the dendrimer aggregation pattern and their antimicrobial activity. In this respect, it is clear that peptide-based dendrimers need to satisfy similar requirements as antimicrobial peptides for driving the interaction to biological membranes in terms of hydrophobicity (end-tagging hydrophobic moieties) and charge distribution (for a recent review see [1]). However, the branched structure of dendrimeric molecules gives them improved properties as compared to linear peptides in general. This includes higher stability against proteolysis, increased solubility, decreased toxicity to human cells [18] and lower MIC [19].

The reason for dendrimer antimicrobial activity does not necessarily have to do with a membrane disrupting mechanism since the dendrimer with lowest MIC did not have the strongest ability to distort membranes. Another parameter to consider is the net charge and the fluidity of the lipid bilayer. It has been found that dendrimer adsorption can also lead to permeability changes of the membrane and thus affect cell life without inducing major cell membrane restructuring [20]. On the other hand, the membrane disturbing properties are somewhat correlated with the hemotoxicity expressed by these dendrimers (Table 1). The most harmful for red blood cell membranes is compound D101. Dendrimer D100 is two times less hemotoxic, whereas dendrimer D103 with the lowest antimicrobial activity expresses approximately 25% hemotoxicity at 124 μM concentration (it precipitates at higher concentration). POPC was used in this study because it is one of the most abundant lipids of mammalian cells and it is known to form supported lipid bilayers of high quality [21]. Formation of bilayers of bacterial lipid extracts is not straightforward since they contain a higher content of lipids that either carry a negative charge or induce high curvatures such as phosphatidylglycerol and cardiolipin respectively. We, however, have recently developed optimized methods to deposit total lipids extracts from *E. coli* on solid supports using vesicle fusion (manuscript in preparation [22]). POPC membranes are mimics closer to mammalian than bacterial cell membranes, and the development of *E. coli* natural membrane mimics will certainly be valuable tools in order to further validate our results into bacterial mimics.

## 3. Experimental Section

### 3.1. Materials

1-Palmitoyl-2-oleoyl-*sn*-glycero-3-phosphocholine (POPC) was purchased from Avanti Polar Lipids, Inc. (Alabaster, AL, USA) and used without further purification. l-Lysine was purchased from IRIS Biotech gmbH (Marktredwitz, Germany). *N,N*'-Dicyclohexylcarbodiimide (DCC), hydroxybenzotriazol (HOBt), *N*-hydroxysuccinimide (HOSu), chloroform, *N,N*-dimethylformamide (DMF), methyl alcohol (MeOH), ethylenediamine, sodium phosphate monobasic (Na_2_HPO_4_), sodium phosphate dibasic (NaH_2_PO_4_) and sodium chloride (NaCl) were purchased from Sigma Aldrich Inc. (St. Louis, MO, USA). Ultrapure Milli-Q (MQ) water with a resistivity of 18.2 MΩ (Branstead Nanopure 7145, Thermo Fisher Scientific Inc., (Waltham, MA, USA) was used for all cleaning procedures and for sample and buffer preparation. Hellmanex 2% (Hellma GmbH & Co, Müllheim, Germany) and absolute ethanol were used for cleaning QCM-D sensor crystals purchased from Q-Sense AB (Västre Frölunda, Sweden). All solvents and reagents were of analytical grade and were used without further purification.

### 3.2. Dendrimer Synthesis

Mass spectra were recorded with a Mariner ESI time-of-flight mass spectrometer (Applied Biosystems/PerSeptive Biosystems, Inc., Framingham, MA, USA) for the samples prepared in MeOH. The proton and carbon NMR spectra were recorded using a Bruker Avance spectrometer (Bruker BioSpin GMBH, Rheinstetten, Germany) at 500 or 400 MHz, respectively, using deuterated solvents and TMS as an internal standard. Chemical shifts are reported as δ values in parts per million, and coupling constants are given in hertz. Thin layer chromatography (TLC) was performed on aluminum sheets with silica gel 60 F_254_ from Merck (Darmstadt, Germany). Column chromatography (CC) was carried out using silica gel (230–400 mesh) from Merck or Sephadex LH20. The TLC spots were visualized by treatment with 1% alcoholic solutions of ninhydrin and heating.

*N,N'-Tetrakis(methoxycarbonylethyl)-l-lysine* (**1**). Initially, l-lysine (0.1 mol) was dispersed in MeOH (150 mL), followed by NaOH (4 g, 0.1 mol) in MeOH (300 mL) and methyl acrylate (51.65 g, 0.6 mol, 54 mL). The reaction mixture was heated at its boiling point for 48 h and then cooled down. Vacuum was used to remove the solvent and the oily residue was dispersed in acetone (300 mL) and 1 M HCl in MeOH (100 mL). The solution was filtered and then evaporated to dryness. The product was purified by flash silica gel chromatography with EtOAc/Hex (8:2) + 5% MeOH, giving 23.9 g (48.8%) of *N,N*'-tetrakis(methoxycarbonylethyl)-l-lysine as a colorless oil. ESI LRMS (MeOH): 491 [M+H]^+^, 513 [M+Na]^+^. ^1^H-NMR (500 MHz, CDCl_3_) δ 1.34 (m, 2H, γCH_2_), 1.49 (m, 2H, δCH_2_), 1.59, 1.78 (2m, 2H, βCH_2_), 2.44–2.53 (bm, 10H, α, εC*H*_2_COOMe, εCH_2_), 2.86, 2.97 (2m, 8H, α, εN-C*H*_2_-C), 3.26 (t, *J =* 7.1 Hz, 1H, αCH), 3.66 (2s, 12H, OCH_3_). ^13^C-NMR δ 24.6 (γC), 26.1 (δC), 28.6 (βC), 31.6, 33.7 (α, ε*C*H_2_COOMe), 47.0, 48.6 [α, εN-(CH_2_)_2_], 51.5, 51.6 (OCH_3_), 53.1 (εC), 63.8 (αC), 172.6, 172.7 (COOMe), 175.9 (COOH). [D]^25.9 °C^ = −33.81 (*c* 2, acetone).

*N,N'-Tetrakis(methoxycarbonylethyl)-l-lysine tryptamide* (**2**). At 0 °C, *N,N'*-tetrakis(methoxy-carbonylethyl)-l-lysine (10 mmol) was dissolved in DMF (30 mL), tryptamine (3.2 g, 20 mmol), HOBt monohydrate (1.54 g, 10 mmol,) and DCC (2.1 g, 10.17 mmol) were added successively. This mixture was then stirred at 0 °C for 1 h and further stirred at room temperature for 24 h. Precipitated dicyclohexyl urea (DCU) was isolated by filtration and the filtrate was concentrated under vacuum. The oily residue was then dissolved in EtOAc (150 mL) and washed by successively with 150 mL of 10% aqueous Na_2_CO_3_, water, 1% citric acid (three times) and brine. The organic layer was separated and dried over anhydrous MgSO_4_. The residue was filtrated and evaporated to dryness, and then purified by column chromatography on silica gel eluting with EtOAc/Hex (7:3) + 5% MeOH, to afford 5 g (79%) of title compound **2** as a dark orange oil. ESI LRMS (MeOH): 633 [M+H]^+^, 655 [M+Na]^+^. ^1^H-NMR (500 MHz, CDCl_3_) δ 1.2–1.8 (3m, 6H, γ, δ, βCH_2_), 2.25–2.45 (bm, 10H, C*H*_2_COOMe, εCH_2_), 2.75 (m, 8H, α, εN-C*H*_2_-C), 2.99 (t, *J =* 7.0 Hz, 2H, CH_2_-Ar *trNH*), 3.05 (dd, *J =* 2.4, 5.2 Hz, 1H, αCH), 3.5–3.7 (m, 14H, -C*H*_2_-NH *trNH*, OCH_3_), 7.04 (s, 1H, C_1_ *trNH*), 7.09, 7.16 (2 m, 2H, C_5_, C_6_ *trNH*), 7.34 (d, *J =* 8.1 Hz, 1H, C_7_ *trNH*), 7.62 (d, *J =* 7.8 Hz, 1H, C_4_ *trNH*). ^13^C-NMR δ 25.2 (*C*H_2_-Ar *trNH*), 25.8, 26.7, 27.3 (γ, δ, βCH_2_), 32.4, 33.3 (α, ε*C*H_2_COOMe), 39.4 (*C*H_2_-NH *trNH*), 46.4, 49.2 [α, εN-(CH_2_)_2_], 51.4, 51.5 (OCH_3_), 53.5 (εC), 64.8 (αC), 111.1, 113.0, 118.7, 119.1, 121.8, 121.9, 127.4, 136.4 (C_7_, C_2_, C_4_, C_5_, C_6_, C_1_, C_3_, C_8_ *trNH*), 172.7 (COOMe), 173.0 (CONH).

*N,N'-Tetrakis(aminoethylaminocarbonylethyl)-l-lysine tryptamide* (**3**). Compound **2** (5 mmol) was dissolved in MeOH (20 mL) and added dropwise to a mixture of ethylenediamine (22.3 g, 25 mL) and MeOH (50 mL) cooled to 0 °C. The reaction was equilibrated at room temperature and stirred for 5 days. The solvent was evaporated to dryness and then the residue was mixed with *n*-butanol (20 mL) and evaporated again. The final compound, after being dried under reduced pressure was used without further purification (3.68 g, yield 99%). ESI LRMS (MeOH): 745 [M+H]^+^, 767 [M+Na]^+^. ^1^H-NMR (500 MHz, DMSO-D_6_) δ 1.2–1.6 (4m, 6H, γ, δ, βCH_2_), 2.19 (m, 8H, C*H*_2_CONH), 2.3 (m, 2H, εCH_2_), 2.50–3.05 (4bm, 27H, α, εN-C*H*_2_-C, HN-C*H*_2_-C*H*_2_-NH, CH_2_-Ar *trNH*, αCH), 3.35 (m, 2H, -C*H*_2_-NH *trNH*), 6.96, 7.04 (2 m, 2H, C_6_, C_5_ *trNH*), 7.12 (s, 1H, C_1_ *trNH*), 7.32 (d, *J =* 8.1 Hz, 1H, C_7_ *trNH*), 7.54 (d, *J =* 7.8 Hz, 1H, C_4_ *trNH*), 7.9–8.1 (m, 8H, NH_2_). ^13^C-NMR δ 22.5 (γC), 25.3 (*C*H_2_-Ar *trNH*), 26.7 (δC), 28.2 (βC), 33.3, 34.8 (ε, α*C*H_2_CONH), 39.3 (-*C*H_2_-NH *trNH*), 41.3, 42.2 (NH-*C*H_2_-*C*H_2_-NH), 46.8, 49.5 [α, εN-(CH_2_)_2_], 52.8 (εC), 63.3 (αC), 111.3, 111.7, 118.1, 118.2, 120.8, 122.6, 127.1, 136.2 (C_7_, C_2_, C_4_, C_5_, C_6_, C_1_, C_3_, C_8_ *trNH*), 171.4, 171.5 (*C*ONH-(CH_2_)_2_-NH_2_), 171.9 (CONH).

**Scheme 1:**
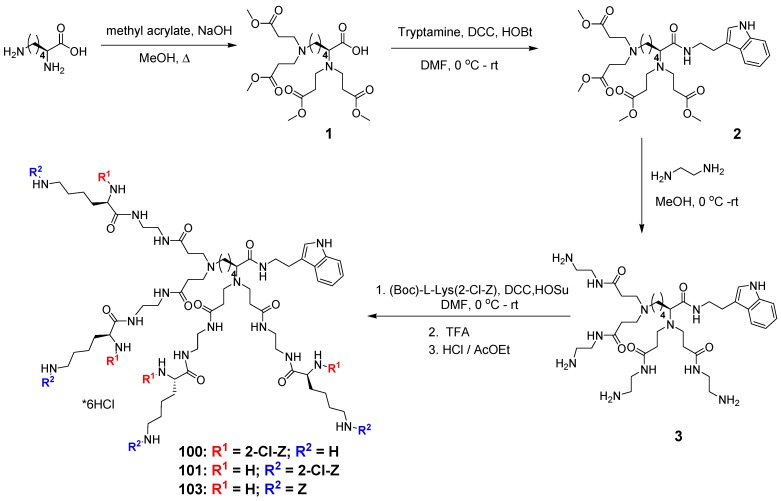
Synthesis route for dendrimer D100, D101 and D103.

Synthesis of dendrimers **4***.* The dendrimers were synthesized as outlined in Scheme 1. First, the core compound **3** (1.86 g, 2.5 mmol) was dissolved in DMF (20 mL). To the obtained mixture, a solution of (2-Cl-Z)-Lys(Boc)-OH or Boc-Lys(2-Cl-Z)-OH (4.56 g, 11 mmol) or Z-Lys(Boc)-OH or Boc-Lys(Z)-OH (4.18 g, 11 mmol), 1.26 g of HOSu (11 mmol) and 2.31 g (11 mmol) of DCC in THF (20 mL) then added and stirred for 48 h at room temperature. Precipitated DCU was isolated by filtration and the filtrate was concentrated in vacuum. The residue was dissolved in EtOAc (150 mL) and washed successively with 10% aqueous Na_2_CO_3_, water, 1% citric acid solution (150 mL of each). The organic layer was separated and dried over anhydrous MgSO_4_, then filtered and evaporated to dryness. The crude dendrimeric compounds was purified on Sephadex LH-20 to provide the products as amorphous foam. Then the dendrimers were converted to their hexahydrochlorides by deprotection of Boc groups with TFA and replacement of trifluoroacetate counter-ions with chloride anion using of HCl-saturated AcOEt, to afford the respective dendrimers D100–103.

*Dendrimer* D100. C_92_H_132_O_17_N_20_Cl_4_·6HCl, M = 2,150.7 g/mol; Yield 1.1g (62.8%), brown hygroscopic foam. ESI LRMS (MeOH): 643.7 [M+3H, *main signal*]^3+^, 661.7 [M+2H+Na+MeOH]^3+^, 679.7 [M+H+2Na+2MeOH]^3+^, 965 [M+2H]^2+^, 995 [M+Na+K]^2+^. ^1^H-NMR (400 MHz, DMSO-D_6_, 298K) δ 1.2–1.7 (bm, 30H, γ, δ, βCH_2_ *lysines*, β, γ, δCH_2_ *core*), 2.1–2.4 (2m, 10H, C*H*_2_CONH, εCH_2_ *core*), 2.6–2.9 (2m, 8H, α, εN-C*H*_2_-C), 3.0–3.45 (2m, 29H, εCH_2_ *lysines*, HN-C*H*_2_-C*H*_2_-NH *core*, CH_2_-Ar *trNH*, C*H*_2_-NH *trNH*, αCH *core*), 3.9 (m, 4H, αCH *lysines*), 5.05–5.2 (4s, 8H, Ar-C*H*_2_O), 6.96, 7.05, 7.14 (3m, 3H, C_1_, C_5_, C_6_ *trNH*), 7.29–7.36 (bm, 9H, C_4_H, C_5_H *2-Cl-Z*, C_7_ *trNH*), 7.40–7.50 (2m, 8H, C_3_H, C_6_H *2-Cl-Z*), 7.55 (m, 1H, C_4_ *trNH*). ^13^C-NMR δ 22.4 (γC *lysines*), 23.4 (γC *core*), 24.6 (δC *core*), 25.0 (*C*H_2_-Ar *trNH*), 26.2 (βC* core*), 29.0 (δC *lysines*), 31.2 (βC *lysines*), 33.4, 34.6 (ε, α*C*H_2_CONH *core*), 38.1, 38.2 (εC *lysines*), 39.1 (-*C*H_2_-NH *trNH*), 39.8 (NH*C*H_2_*C*H_2_NH *core*), 46.7, 49.1 [α, εN-(CH_2_)_2_ *core*], 51.6 (εC *core*), 54.5 (αC lysines), 62.4 (Ar-*C*H_2_O), 63.3 (αC *core*), 111.0, 111.7, 117.8, 117.9, 120.4, 122.2 (C_7_, C_2_, C_4_, C_5_, C_6_, C_1_ *trNH*), 126.8 (C_5_ *2-Cl-Z*), 126.9 (C_3_ *trNH*), 128.8, 129.0, 129.1, 131.8, 134.1 (C_6_, C_4_, C_3_, C_2_, C_1_ *2-Cl-Z*), 136.1 (C_8_ *trNH*), 155.0 (O-CO-NH), 170.7, 170.9 (CH_2_*C*ONH *core*), 171.1 (CONH *lysines*), 171.5 (CONH *core*).

*Dendrimer* D101. C_92_H_132_O_17_N_20_Cl_4_·6HCl, M = 2,150.7 g/mol. Yield 1.1 g (66.7%), yellow hygroscopic foam. ESI LRMS **(**MeOH**)**: 587.7 [M-*2-Cl-Z*+3H]^3+^, 643.7 [M+3H, *main signal*]^3+^, 661.7 [M+2H+Na+MeOH]^3+^, 965 [M+2H]^2+^, 995 [M+Na+K]^2+^. ^1^H-NMR (400 MHz, DMSO-D_6_, 298K) δ 1.3–1.7 (bm, 30H, γ, δ, βCH_2_ *lysines*, γ, δ, βCH_2_ *core*), 2.15–2.4 (2m, 10H, C***H***_2_CONH, εCH_2_ *core*), 2.5–2.9 (2m, 8H, α, εN-C***H***_2_-C), 3.0–3.5 (2m, 29H, εCH_2_ *lysines*, HN-C***H***_2_-C***H***_2_-NH *core*, CH_2_-Ar *trNH*, -C***H***_2_-NH *trNH*, αCH *core*), 3.84 (m, 4H, αCH *lysines*), 5.09–5.2 (4s, 8H, Ar-C***H***_2_O), 6.95, 7.07, 7.14 (3m, 3H, C_1_, C_5_, C_6_ *trNH*), 7.30–7.36 (bm, 9H, C_4_H, C_5_H *2-Cl-Z*, C_7_ *trNH*), 7.40–7.50 (2m, 8H, C_3_H, C_6_H *2-Cl-Z*), 7.54 (m, 1H, C_4_ *trNH*). ^13^C-NMR δ 22.3 (γC *lysines*), 23.5 (γC *core*), 24.8 (δC *core*), 25.1 (***C***H_2_-Ar *trNH*), 26.3 (βC *core*), 28.9 (δC *lysines*), 31.1 (βC* lysines*), 33.5, 34.7 (ε, α***C***H_2_CONH *core*), 38.0, 38.1 (εC *lysines*), 39.1 (-***C***H_2_-NH *trNH*), 39.9 (NH***C***H_2_***C***H_2_NH *core*), 46.6, 49.2 (α, εN-(CH_2_)_2_ *core*), 51.5 (εC *core*), 54.2 (αC *lysines*), 62.2 (Ar-***C***H_2_O), 63.1 (αC (*core*), 111.0, 111.7, 117.8, 117.9, 120.4, 122.1 (C_7_, C_2_, C_4_, C_5_, C_6_, C_1_ *trNH*), 126.9 (C_5_ *2-Cl-Z*), 127.0 (C_3_ *trNH*), 154.8 (O-CO-NH), 170.5, 170.8 (CH_2_***C***ONH *core*), 171.2 (CONH *lysines*), 171.4 (CONH *core*).

*Dendrimer* D103. C_92_H_136_O_17_N_20_ × 6HCl, M = 2,012.9 g/mol. Yield 1.0 g (68.4%), brown hygroscopic foam. ESI LRMS (MeOH): 509 [M−2 × *Z*+3H]^3+^, 553.7 [M−*Z*+3H]^3+^, 598.3 [M+3H, * main signal*]^3+^, 616.3 [M+2H+Na+MeOH]^3+^, 897 [M+2H]^2+^.^1^H-NMR (400 MHz, DMSO-D_6_, 298K) δ 1.2–1.7 (4m, 30H, γ, δ, βCH_2_ *lysines*, β, γ, δCH_2_ *core*), 2.1–2.4 (bm, 10H, C*H*_2_CONH, εCH_2_ *core*), 2.5–2.9 (2m, 8H, α, εN-C*H*_2_-C), 3.0–3.36 (3m, 29H, εCH_2_ *lysines*, HN-C*H*_2_-C*H*_2_-NH *core*, CH_2_-Ar *trNH*, C*H*_2_-NH *trNH*, αCH *core*), 3.9 (m, 4H, αCH *lysines*), 5.07–5.2 (4s, 8H, Ar-C*H*_2_O), 6.96, 7.05, 7.13 (2m, 3H, C_1_, C_5_, C_6_ *trNH*), 7.23–7.4 (m, 21H, Ar-H *Z*, C_7_* trNH*), 7.54 (m, 1H, C_4_ *trNH*). ^13^C-NMR δ 22.3 (γC *lysines*), 23.4 (γC *core*), 24.3 (δC *core*), 25.0 (*C*H_2_-Ar *trNH*), 26.7 (βC* core*), 29.0 (δC *lysines*), 31.1 (βC *lysines*), 33.7, 34.3 (ε, α*C*H_2_CONH *core*), 38.0, 38.1 (εC *lysines*), 39.1 (*C*H_2_-NH *trNH*), 39.9 (NH*C*H_2_*C*H_2_NH *core*), 46.5, 48.7 [α, εN-(CH_2_)_2_ *core*], 51.6 (εC *core*), 54.3 (αC *lysines*), 63.2 (αC *core*), 65.4 (Ar-*C*H_2_O), 111.0, 111.6, 117.8, 117.9, 120.5, 122.2, 127.0 (C_7_, C_2_, C_4_, C_5_, C_6_, C_1_, C_3_ *trNH*), 127.1, 127.3, 127.9 (C_2_, C_3_, C_4_, C_5_, C_6_ *Z*), 136.1 (C_8_ *trNH*), 136.9 (C_1_ *Z*), 155.3, 155.5 (O-CO-NH), 170.7 (CH_2_*C*ONH *core*), 171.2 (CONH *core*), 171.4 (CONH *lysines*), 171.4 (CH_2_*C*ONH *core*), 171.9 (CONH *core*), 172.1 (CONH *lysines*).

### 3.3. Dendrimer Solutions

Prior to use dendrimers were dissolved in small quantities of methanol. For QCM-D experiments, the dendrimers were diluted into 10 mM phosphate buffered saline (PBS) containing 100 mM NaCl at pH 7.4 in concentrations ranging from 1 μM to 50 μM. In all experiments dendrimers were bath sonicated (Branson 1510, Branson Ultrasonics Corporation, Danbury, CT, USA) prior to use in order to eliminate kinetically trapped self-aggregates formed in solution.

### 3.4. Small Unilamellar Vesicles (SUVs)

Lipids dissolved in chloroform were dried under a soft stream of nitrogen onto the walls of clean glass vials. The films were left on vacuum overnight in order to remove any remaining organic solvent and stored at −18 °C until use. Lipid films were re-suspended in MQ water to a concentration of 0.5 mg/mL and were left to hydrate for minimum one hour at room temperature. SUVs were prepared by tip sonication of the suspensions until clarity. Sonication was carried out on a 50% duty cycle (5 s on followed by 5 s off) in order to reduce the heat produced. The lipid solutions were diluted to 0.1 mg/mL prior to use.

### 3.5. Dissipation-Enhanced Quartz Crystal Microbalance (QCM-D)

Experiments were performed with a Q-SENSE E4 system (Biolin Scientific AB, Stockholm, Sweden). The quartz sensor crystals were coated with 50 nm silicon dioxide purchased from Q-Sense. For cleaning, O-rings and the sensor surfaces were placed in 2% Hellmanex for 10 min followed by thorough rinsing in absolute ethanol and ultrapure water. They were then dried in a stream of nitrogen and the surfaces were subsequently oxidized in a UV-ozone chamber (BioForce Nanosciences, Inc., Ames, IA, USA) for 10 min in order to remove any organic contamination. Before performing any measurements the instrument was equilibrated at 25 °C until a stable baseline in water was recorded. The isotherms for dendrimer adsorption to clean silica were obtained using dendrimer solutions of concentrations ranging from 1 to 50 µM. For all concentrations, the adsorption was monitored for 1 h under a constant flow of 100 µL/min. For bilayer formation by vesicle fusion, lipids were pumped into the sample cells at a concentration of 100 μg/mL. After successful supported lipid bilayer formation, the membranes were rinsed with PBS before addition of dendrimers. Data corresponding to the 7th overtone are shown in this work. Data for D101 was previously published in ref. [12], and has been redrawn here for comparison to D100 and D103.

### 3.6. Determination of Antimicrobial Activity

Bacteria *Staphylococcus aureus* ATCC 25923, *Staphylococcus aureus* ATCC 43300,* Escherichia c*oli ATCC 25922 and *Pseudomonas aeruginosa* ATCC 27853 were cultivated on tryptone—soy agar (TSA; Oxoid) for 24 h at 37 °C. Broth microdilution susceptibility test was performed as described in Committee Laboratory Standards (CLSI) reference method M07-A8 [23]. A series of the twofold dendrimer dilutions in DMSO and twofold polymyxin B and penicillin G dilutions in cation-adjusted Mueller-Hinton broth (CAMHB) were diluted 1:94 with CAMHB. Then, 95 µL aliquots were dispensed into microdilution sterile plates (Mar-Four, Lodz, Poland) followed by the addition of 5 µL of bacteria inoculum containing 10^6^ CFU/mL. The final concentration of dendrimer ranged from 256 to 2 µg/mL (or ~100–~3 µM), polymyxin B and penicillin G from 8–0.15 µg/mL (that corresponds to 5.8 to 0.1 µM and 21.5 to 0.4 µM, for polymyxin B and penicillin G, respectively), all in twofold dilution steps. The plates were incubated at 35 °C and were read after 18 or 24 h depending on the bacterial strain. The minimal inhibitory concentration (MIC) was defined as the lowest drug concentration that reduced the growth by 100%.

Dendrimer-induced hemolysis was measured according to previously reported protocol [24]. Briefly, fresh human red blood cells (RBCs, from healthy volunteers), were suspended in phosphate buffered saline (PBS, pH 7.4). The prepared suspensions of 1% hematocrit were incubated with serial concentration of dendrimers at 23 °C for 30 min. The absorbance of the supernatant obtained after centrifugation (4000 rpm, 5 min) was measured at 540 nm (Spectrostar Omega, BMG Labtech Ortenberg, Germany). RBCs incubated with PBS and double-distilled water (30 min at 23 °C) were used as indicators of 0 and 100% hemolysis, respectively. The hemotoxicity (HEM) was calculated as follows:

(1) $$HEM\;[\%]=\frac{A-A_{b}}{A_{100\%}-A_{c}}$$

where A: Absorbance of the samples incubated with dendrimers; A_b_: Absorbance of the blank samples; A_100%_: Absorbance of the reference; A_c_: Absorbance of red blood cells in PBS, hematocrit 1%.

## 4. Conclusions

Dendrimers of varying chemical structure were synthesized and their antimicrobial activity against several bacterial strains was determined. Since these dendrimers are expected to act in a non-specific manner and target the cellular membrane, their adsorption affinity at negatively charged silica surfaces and on zwitterionic lipid bilayers was studied, as well as their capacity to significantly rearrange the lipid bilayer structure by QCM-D. The data suggests that the supramolecular structure in terms of charge distribution and amphiphilicity, rather than net charge, is the main driver for major disruption of cellular membranes and this correlates well with hemolytic activity rather than antimicrobial activity of these compounds.
